# Albumin evokes Ca^2+^-induced cell oxidative stress and apoptosis through TRPM2 channel in renal collecting duct cells reduced by curcumin

**DOI:** 10.1038/s41598-019-48716-x

**Published:** 2019-08-27

**Authors:** Mustafa Nazıroğlu, Bilal Çiğ, Yener Yazğan, Gerburg K. Schwaerzer, Franziska Theilig, László Pecze

**Affiliations:** 1 Neuroscience Research Center, Suleyman Demirel University, Isparta, Turkey; 2 Department of Biophysics, Faculty of Medicine, Suleyman Demirel University, Isparta, Turkey; 3 Department of Neuroscience, Health Science Institute, Suleyman Demirel University, Isparta, Turkey; 4 Institute of Anatomy, Christian-Albrechts-University of Kiel, Kiel, Germany; 5 Anatomy, Department of Medicine, University of Fribourg, Fribourg, Switzerland; 6 Independent Scientist, Neuchhatel, Switzerland

**Keywords:** Cell biology, Molecular medicine

## Abstract

In proteinuric nephropathies of chronic kidney disease, the epithelial cells of the nephron including the collecting duct are exposed to high concentrations of luminal albumin. Albumin is taken up from collecting duct cells by endocytosis causing excessive reactive oxygen species (ROS) production and a proinflammatory response. Curcumin used in the traditional medicine possesses anti-inflammatory and antioxidant effects. ROS and ADP-ribose (ADPR) activate the cation channel TRPM2. We hypothesize, that albumin-induced cell stress and proinflammatory response are mediated by Ca^2+^ and can be reduced by curcumin. The cortical collecting duct (CCD) cells mpkCCD_c14_ exhibit spontaneous and inducible Ca^2+^ oscillations, which can be blocked by pre-treatment with curcumin. Curcumin accumulates in plasma membrane and intracellular vesicles, where it interferes with TRPM2 and decreases the influx of Ca^2+^. Albumin reduces cell viability and increases apoptosis, NF-κB activation, and mitochondrial membrane depolarization via Ca^2+^-dependent signaling, which results in increased ROS production. Albumin-induced cell stress is diminished by the inhibition of TRPM2 after administration of curcumin and ADPR (PARP1) inhibitors. Curcumin did not reduce the Ca2+ elevation induced by thapsigargin in Ca^2+^-free medium, but it reduced the function of store-operated Ca^2+^ channels and ATP-evoked Ca^2+^ response. In conclusion, albumin-induced oxidative stress is mediated by Ca^2+^-dependent signaling via TRPM2 and leads to cell damage and a proinflammatory response, strengthening the role of CCD cells in the progression of chronic kidney disease.

## Introduction

Albumin functions primarily as a carrier protein for fatty acids, and steroids in the blood. In healthy human adults, approximately seven grams of albumin are daily filtered by the glomeruli and partly reabsorbed by epithelial cells via receptor-mediated, clathrin-dependent endocytosis in proximal tubules^1^. Increased urinary albumin excretion is caused by increased glomerular filtration, glomerular damage or tubular injury. Albuminuria is a well-known indicator for renal diseases and is involved in the progression of chronic kidney disease^2^. During diabetic nephropathy increased urinary albumin excretion was detected before changes in serum creatinine levels were observed^3^. With saturation of proximal tubular endocytosis, albumin reaches the tubular lumen of collecting ducts where it is reabsorbed by cortical collecting duct (CCD) cells. Albuminuria induces a proinflammatory response in CCDs promoting chronic kidney disease (CKD)^4^. Exposure of murine CCD cells to albumin induces NF-κB activation, the expression of transforming growth factor-β1 (TGF-β1) and the upregulation of profibrotic signaling markers^4^. Increased oxidative stress and reactive oxygen species (ROS) are frequently found in CKD patients^5^. The current therapeutic approaches slow down the progression of CKD through normalization of blood pressure and maintaining the normal glucose and insulin levels^6^. Therefore, the developments of novel therapies are highly needed to prevent the progression of CKD and improve renal function. Interestingly, new therapeutics might be natural products as curcumin with proven safety profiles^7^.

Turmeric (Curcuma longa) is a popular spice that has been used for centuries in traditional medicine for the treatment of various diseases^8^. Curcumin is the main compound present in turmeric and has been shown to possess a broad spectrum of biological actions including anti-inflammatory, antioxidant, anticarcinogenic, anti-mutagenic, anti-coagulant and anti-infective effects^8,9^. Since pathophysiology of CKD involves oxidative stress and inflammation^10^, the potential beneficial effect of curcumin consumption on the renal diseases have been extensively studied^7^. Nevertheless, the precise mechanisms how proteinuria induces oxidative stress in the CCD cells have not been satisfactorily elucidated so far.

Elevated levels of ROS lead to oxidative stress and damage lipids, proteins and DNA, and are linked to a myriad of pathologies^11^. Oxidative stress can disrupt normal physiological pathways and cause cell death, a process largely mediated through Ca^2+^ signaling^12^. Oxidative stress induces Ca^2+^ influx from the extracellular environment into the cytoplasm through transient receptor potential (TRP) channels and the store-operated Ca^2+^ channels (SOCE)^13,14^. TRPM2 channel was the first identified TRP channel, which is sensitive to ROS. It is suggested that hydrogen peroxide triggers the intracellular production of ADP-ribose (ADPR) and activates TRPM2^15^.

In this study, we show that Ca^2+^ signaling is involved in albumin-induced oxidative stress, when calcium enters the cells via TRPM2 channels. The Ca^2+^ influx via TRPM2 is strongly reduced by cellular pretreatment with curcumin.

## Results

### Establishment of Ca^2+^ oscillations blocked by curcumin

**Figure 1:**
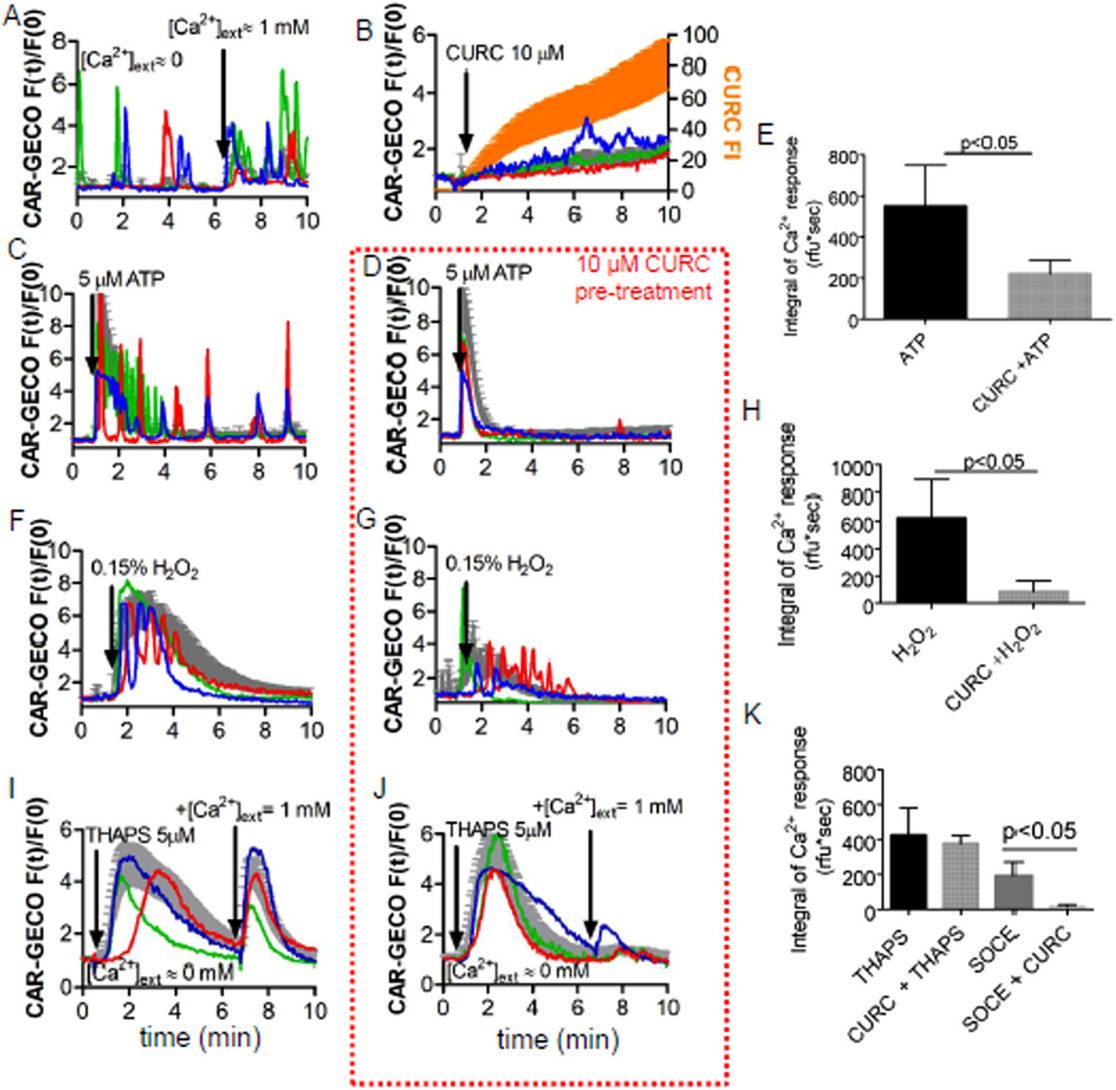
Calcium signaling is influenced by curcumin. (**A**–**D**,**F**,**G**,**I**,**J**) Single-cell (colored traces) and average fluorescence (grey traces) recordings from time-lapse videos show changes in [Ca^2+^]_i_. Bars represent standard deviations (SD). Each figure represents the results of one representative experiment out of three/four with similar results. (**A**) “Spontaneous” Ca^2+^ oscillations. (**B**) curcumin (CURC, 10 µM) uptake slightly decreased the basal [Ca^2+^]_i_, but it did not evoke robust Ca^2+^ oscillations (measured in green channel). Yellow curve represents the time-course of curcumin uptake (measured in green channel). (**C**,**D**) Administration of 5 µM ATP-evoked Ca^2+^ response, which was moderated by curcumin (CURC) pre-treatment (10 µM, 5 min). (**F-G)** Similarly, 0.15% H_2_O_2_ solutions evoked Ca^2+^ responses, but this effect was reduced by curcumin (CURC) pretreatment (10 µM, 5 min). (**I**,**J)** Curcumin did not reduce significantly the Ca^2+^ elevation induced by thapsigargin (THAPS, 5 µM) in Ca^2+^-free medium, but significantly reduced the function of store-operated Ca^2+^ channels (SOCE). (**E**,**H**,**K**) Statistical comparison of the integrals of the evoked Ca^2+^ responses with/without curcumin pretreatment.

Changes in [Ca^2+^]_i_ were monitored with the genetically encoded Ca^2+^ indicator protein CAR-GECO1. Even without any treatments, mpkCCD_c14_ cells showed “spontaneous” Ca^2+^ oscillations observable in 3–40% of cells (Fig. 1A), which are similar to the spontaneous Ca^2+^ oscillations found in isolated CCD due to purinergic signaling^16^. These spontaneous oscillations were present but less intensive in the absence of extracellular Ca^2+^ (Fig. 1A). The total Ca^2+^ oscillations were slightly decreased by the curcumin treatment, because its modulator role on the Ca^2+^ influx in the cells. However, the red fluorescence is slightly increased, which can be caused by the overlap of the curcumin staining and the emission of red fluorescence light or by slight elevations in the basal Ca^2+^ concentrations (Fig. 1B). To investigate the impact of curcumin on Ca^2+^-depending signaling, cells were incubated with different stimulants and curcumin. One of the strong inducers of Ca^2+^ oscillations is extracellular ATP acting on the purinergic receptors. Pre-treatment with curcumin significantly inhibited the ATP- evoked signal (550 ± 198 vs. 220 ± 66, unpaired t-test, p < 0.05): it reduced the integrals of the initial Ca^2+^ signals and the number of the oscillating cells from 90–95% to 5–10% (Fig. 1C–E). A similar effect was observed, when cells were exposed to oxidative extracellular milieu. Curcumin (CURC) significantly reduced the peroxide-evoked Ca^2+^ signaling (623 ± 260 vs. 86 ± 76, unpaired t-test, p < 0.05; Fig. 1F–H). The finding that curcumin treatment was able to reduce the effect of different stimuli indicates that curcumin do not act on a specific plasma membrane receptor, rather than it acts somewhere in the common phospho-inositol pathway.

Detailed analysis of oscillation frequencies and amplitudes are presented in the Supplementary material using a MATLAB code written for this purpose. Previously, it was reported that curcumin inhibits SERCA pump^17^. However, curcumin treatment did not significantly reduce the Ca^2+^ signals evoked by the SERCA-inhibitor thapsigargin (424 ± 156 vs. 379 ± 43, One-way ANOVA + posthoc LSD test, p > 0.05), but it significantly inhibited the store-operated Ca^2+^ entry (194 ± 76 vs. 18 ± 7, One-way ANOVA + post hoc LSD test, p < 0.05) (Fig. 1I–K).

### Intracellular localization of curcumin in mpkCCD_c14_ cells

**Figure 2:**
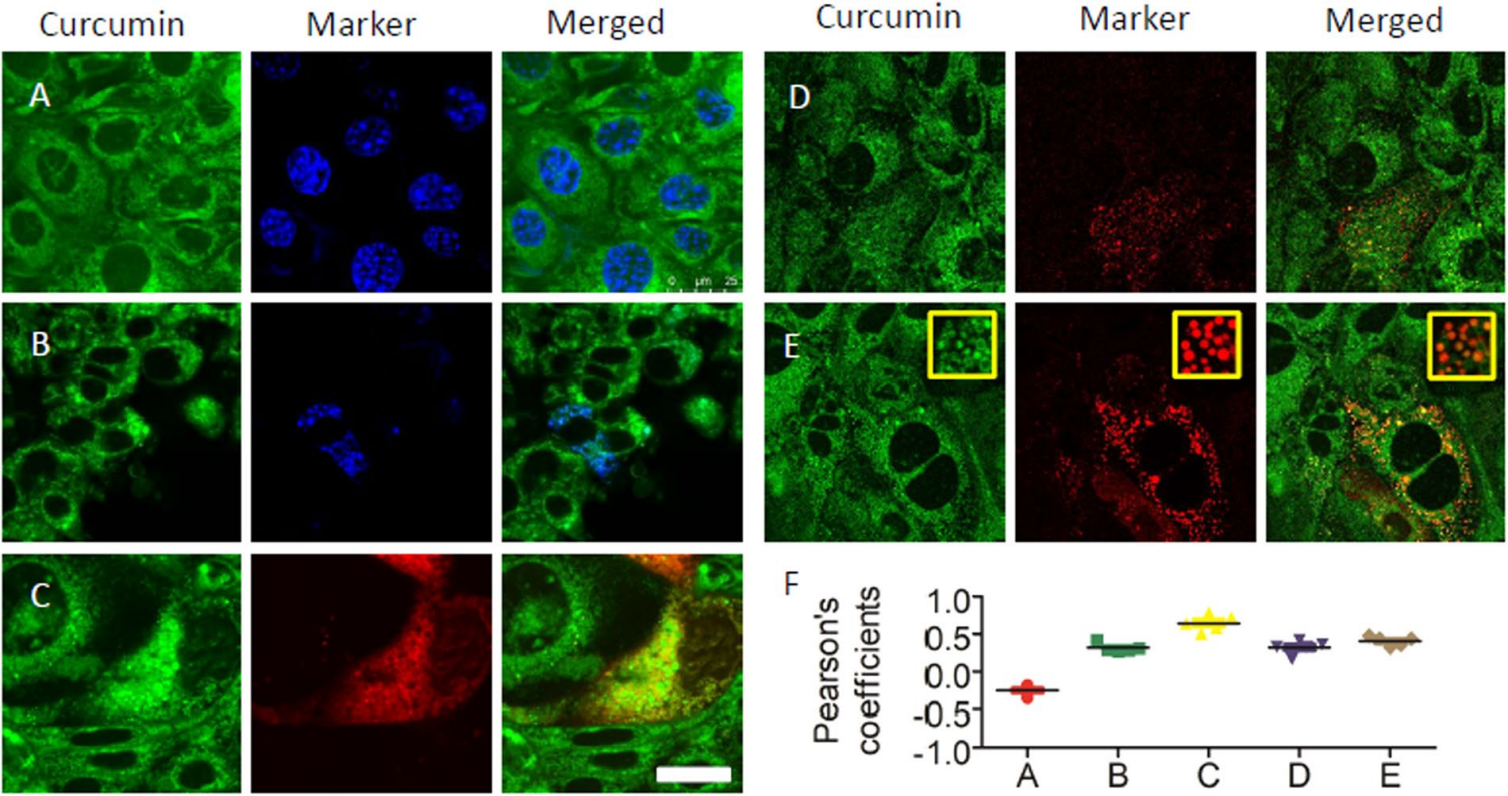
Fluorescence images are showing intracellular localization of curcumin in mpkCCD_c14_ cells. (**A**,**B**) Curcumin staining does not co-localize with nuclei and mitochondria: curcumin (green), nucleus (blue, Hoechst 33342, first row), and mitochondria (blue, mito-BFP, second row). (**C**,**E**) Curcumin is partially present on endoplasmic membranes, clathrin vesicles and lysosomes: Curcumin (green), endoplasmic reticulum (red, m-Cherry-ER-3 third row), clathrin vesicles (red, m-RFP-Clc, first row on the right side) and lysosomes (red, Lamp1-RFP, second row on the right side). Magnification bar represents 25 μm. (**E**) Distribution of Person’s correlation coefficients taking 5 images for each case.

Previously, it had been shown that curcumin stains cells^18^. Therefore, we wanted to know, which cellular organelles are involved in the accumulation of curcumin. Curcumin staining did not co-localize with cell nuclei labeled with blue Hoechst 33342 (Fig. 2A, first row, Pearson’s coefficients = −0.25 ± 0.07, n = 5). Curcumin staining showed weak partial co-localization with mitochondria visualized by mito-BFP (Fig. 2A, second row, Pearson’s coefficients = 0,31 ± 0.06, n = 5) and strong partial co-localization with endoplasmic reticulum (ER) compartments, stained with the red fluorescent protein KDEL having an ER-retention signal (Fig. 2C, third row, Pearson’s coefficients = 0.64 ± 0.11, n = 5). Additionally, visualization of clathrin vesicles and lysosomes, revealed partial co-localization with clathrin vesicles (Fig. 2D, first row on right side, Pearson’s coefficients = 0.32 ± 0.09, n = 5) and lysosomes (Fig. 2E second row on right side, Pearson’s coefficients = 0.40 ± 0.06, n = 5). In some areas, there is a strong co-localization with lysosomes (Insets on Fig. 2E). On Fig. 2F, one can observe the distribution of Pearson’s co-localization coefficients. Pearson’s colocalization coefficients were in the range of + 1 (perfect correlation) to −1 (perfect but negative correlation) with 0, which was indicating the absence of a relationship. Detailed co-localization analysis with other co-localization measures is presented in the Supplementary Material (Supplement 1). The presence of curcumin in membranes and vesicles fits with the site of action of curcumin to inhibit Ca^2+^ entry and the common phospho-inositol pathway and with acting on TRPM2 channels.

### Albumin-induced Ca^2+^ oscillations, cytokine production and NF-κB signaling are inhibited by curcumin

**Figure 3:**
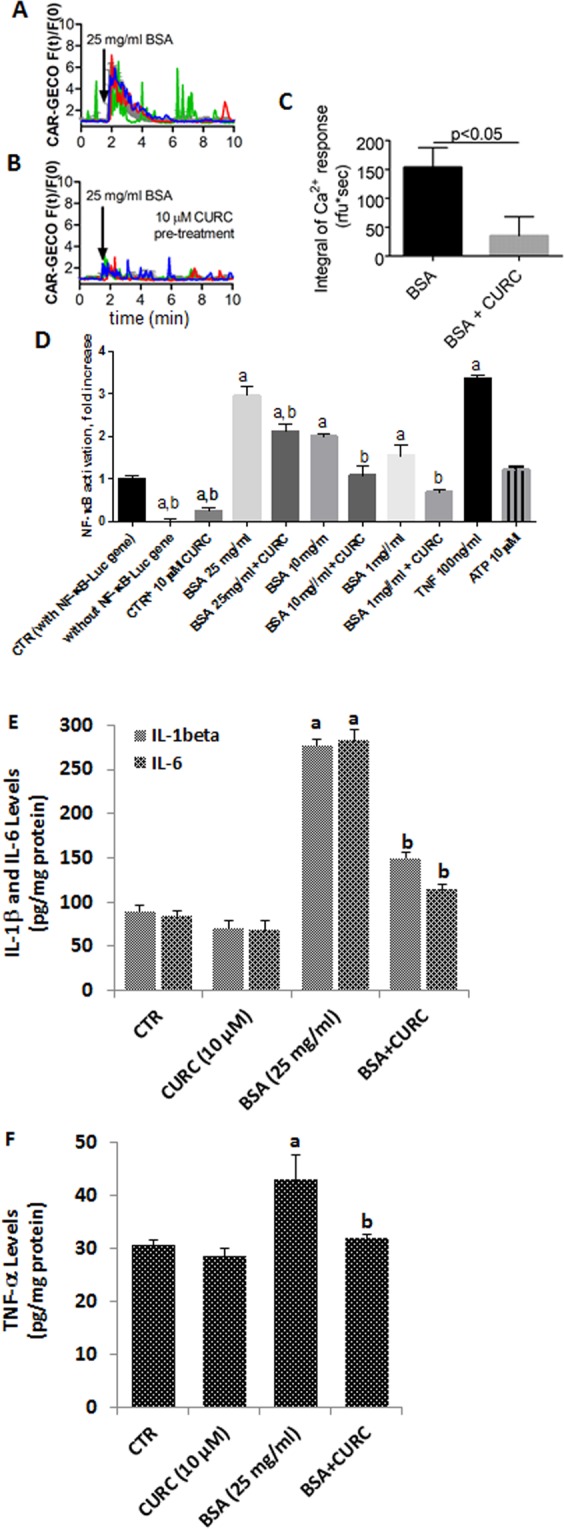
Albumin evokes Ca^2+^ signals, but the NF-κB activity is blocked by curcumin. (**A**,**B)** Single-cell (colored traces) and average fluorescence (gray traces) recordings from time-lapse videos show changes in [Ca^2+^]_i_ after albumin (BSA) administration. Bars represent standard deviations (SD). Experiments were repeated at least three times with similar results. (**C)** Curcumin (CURC) pre-treatment (10 µM) significantly reduced Ca^2+^ signals evoked by albumin. (**D**) Incubation with CURC dose-dependently reduced NF-κB activity evoked by high extracellular protein (BSA) concentration. (**E**,**F**) Incubation with CURC attenuated IL-1β, IL-6 and TNF-α activities evoked by high extracellular protein (BSA), respectively. In Figures A, B, C and D, the letters on the columns denote the following: a - significant difference from control group, One-way ANOVA + post hoc Dunett test, b - significant difference between curcumin-treated and non-treated counterparts. One-way ANOVA + post hoc Sidak’s test. In Figures E and F, ^a^p < 0.05 versus control and CURC groups. ^b^p < 0.05 versus BSA group.

Increased extracellular protein concentration also induced Ca^2+^ oscillations in approximately 70% of the exposed cells. This effect was strongly reduced by pre-treatment of cells with 10 µM curcumin (153 ± 33 vs. 35 ± 32, unpaired t-test, p < 0.05; Fig. 3A–C; Supplementary Material 1). Previous studies have shown that Ca^2+^ oscillations affect NF-κB, tumor necrosis factor-alpha (TNF-α), interleukin 1-beta (IL-1β) and IL-6 activations^19,20^. Thus, we tested how curcumin (10 µM) or decreased extracellular protein concentrations (25 mg/ml, 10 mg/ml, and 1 mg/ml) affect NF-κB activation or TNF-α, IL-1β and IL-6 productions. Control mpkCCD_c14_^NF-κB-luc^ cells show a basal level of NF-κB-activation, which was reduced by administration of 10 µM curcumin into the culture media. Cells which did not express the NF-κB-Luc construct do not produce any light signal. Treatment of mpkCCD_c14_^NF-κB-Luc^ cells with albumin (BSA) for 6 h increased the NF-κB-activation in mpkCCD_c14_^NF-κB-Luc^ cells dose-dependently. In the presence of curcumin, NF-κB activation was significantly diminished. As a positive control, 100 ng/ml of murine TNF-α evoked an intense NF-κB-activation as it was expected. ATP (10 µM) in the extracellular medium, however, did not induce significant NF-κB activation (Fig. 3D). However, TNF-α, IL-1β and IL-6 productions in the mpkCCDc14 cells were increased by albumin treatment, although they were decreased by the curcumin treatment (Fig. 3E,F). These results indicated that there was no interaction between NF-κB activation and intracellular Ca^2+^ oscillations, although albumin induced increase of NF-κB, TNF-α, IL-1β and IL-6 levels were diminished by curcumin treatment.

### Availability of functional TRPM2 channels on the mpkCCD_c14_ and HEK293 cells

**Figure 4:**
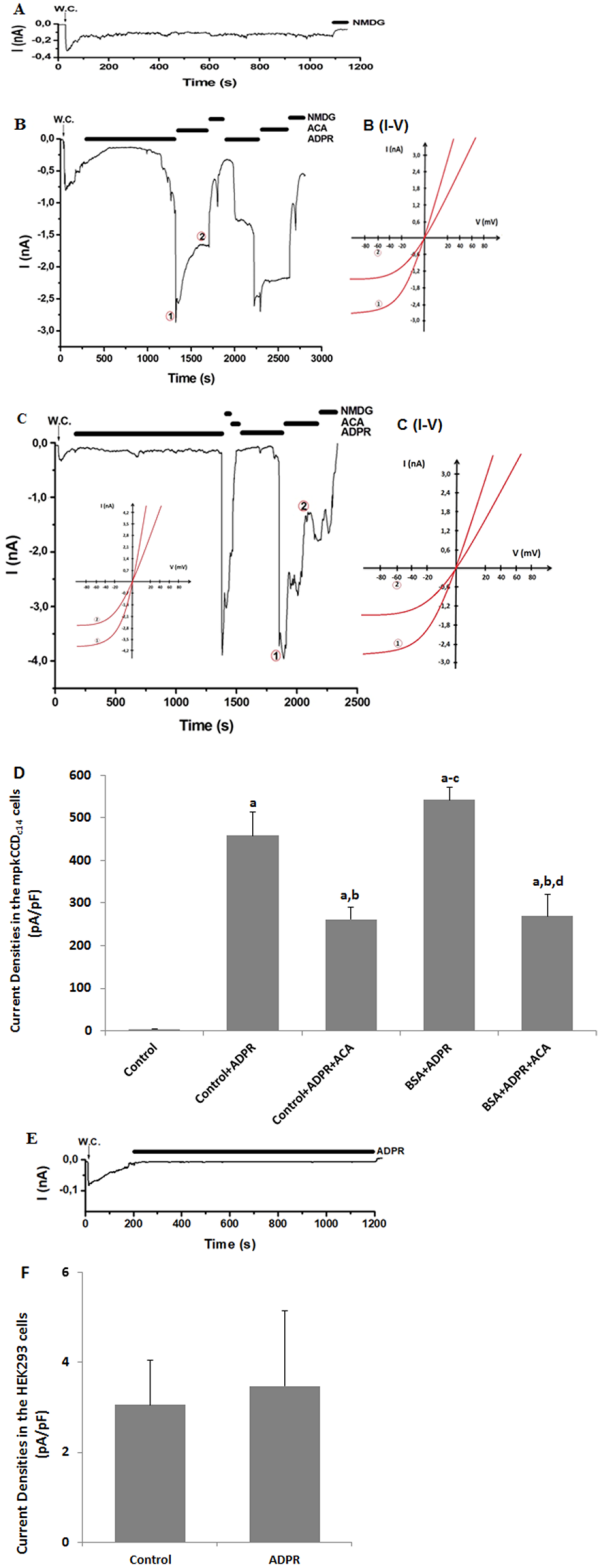
TRPM2 channels are available on mpkCCD_c14_ cells but not in HEK293 cells. The holding potential was set at −60 mV; W.C. denotes whole-cell patch clamp configuration. (**A**) Recordings from a control mpkCCD_c14_ cell without stimulation. (**B)** Recordings from a stimulated mpkCCD_c14_ cell (without BSA treatment). The TRPM2 currents in the cells were stimulated by intracellular (patch pipette) ADPR (1 mM), but the currents were blocked by the extracellular TRPM2 antagonist (ACA and 25 µM) in the patch-chamber. Currents were fully blocked in Na^+^-free solution (NMDG^+^) (**C)** Recordings from a stimulated mpkCCD_c14_ cell (with BSA incubation). The TRPM2 channel was further activated in the cells by intracellular ADPR. (**B**) (I-V) and **C** (I-V) are corresponding I/V relation of currents recorded in (**B**,**C**) at the indicated time points 1 and 2, respectively. (**D)** Current densities of mpkCCD_c14_ cells were calculated by the current amplitudes and cell membrane capacitances. (**E)** TRPM2 records in HEK293 cells. The TRPM2 channel in HEK293 cells was not activated by intracellular ADPR (1 mM in the patch pipette). (**F**) Current densities of HEK293 cells were calculated by the current amplitudes and cell membrane capacitances. Columns represent the mean + SD (n = 6). The letters on the columns denote the following: a - significant difference from control group (p < 0.05). b - significant difference between ACA-treated and non-treated counterparts (p < 0.05). c - significant difference between BSA + ADPR-treated, control + ADPR-treated and non-treated counterparts (p < 0.05). d - significant difference between BSA and BSA + ADPR-treated and non-treated counterparts (p < 0.05).

Results of real time PCR indicated presence of TRPM2 mRNA expression in the medulla, renal cortex and mpkCCDc14 cells (Fig. 3; Supplementary material-1). TRPM2 cation channel has Nudix box domain in C terminal and it is stimulated by intracellular ADPR production^21^. Holding potential in the patch-clamp experiment were kept at −60 mV as described in previous studies^13,21^ and there was no activation of TRPM2 without ADPR stimulation (Fig. 4A). In the presence of intracellular (the patch pipette) ADPR (1 mM), the TRPM2 in mpkCCD_c14_ cells without BSA treatment were gated up to 3.0 nA. N-(p-amylcinnamoyl) anthranilic acid (ACA) and NMDG^+^ are non-specific blockers of TRPM2^13,21^ and the ADPR-induced currents in the cells were reversibly blocked by extracellular ACA and NMDG^+^ treatments (Fig. 4B,D). The ADPR-induced TRPM2 current densities were markedly (p < 0.05) higher in the control + ADPR group than in control, although their denisites were markedly (p < 0.05) diminished in the control + ADPR + ACA group by the ACA treatment. In addition, we observed the TRPM2 activation up to 4.0 nA through intracellular ADPR treatment in the mpkCCD_c14_ cells after BSA treatment (Fig. 4C), although the TRPM2 activation were diminished by extracellular ACA treatment. These results obviously indicated the presence of functioning TRPM2 channel in mpkCCD_c14_ cells and an activating role of oxidant BSA on TRPM2 channel activity.

For confirming the patch-clamp results in the mpkCCD_c14_ cells, the analyses were repeated in the HEK293 cells. It is well known that HEK293 cells do not express TRPM2^22,23^. We observed that ADPR did not induce TRPM2 channel current in the absence of TRPM2 channel in the HEK293 cell line (Fig. 4E,F). Therefore, we further confirmed involvement of TRPM2 channel on mpkCCD_c14_ cells by the HEK293 cell results.

### Albumin (BSA)-induced increase of the intracellular free Ca^2+^ fluorescence intensity in the mpkCCD_c14_ cells was diminished through TRPM2 inhibition by curcumin

**Figure 5:**
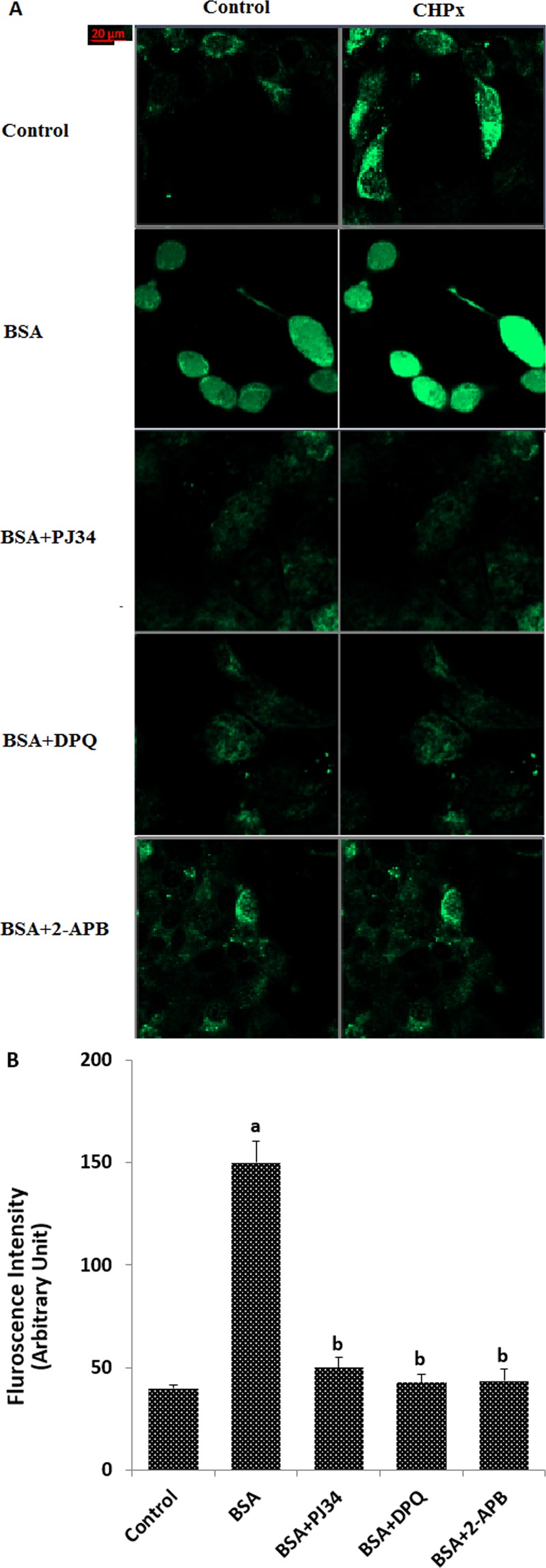
BSA induces TRPM2-dependent increase in the [Ca^2+^]_i_ fluorescence intensity in the mpkCCD_c14_ cells: Modulator role of curcumin (CURC). (**A**) Representatives confocal images showing Fluo-3 (green) in cells under control (CTL) conditions or after exposure to BSA (25 mg/ml), BSA + CURC for 24 h, BSA + PARP-1 inhibitors (30 μM PJ34 and 10 μM DPQ for 30 min prior to BSA, cumene hydroperoxide (CHPx and 1 mM for 10 min) incubations) and TRPM2 channel antagonist (2-APB and 0.1 mM for 30 min before cells were incubated with BSA and CHPx). Details of the incubations and stimulations were given in material and method section. Briefly, Cells in the four groups (BSA, BSA + PJ34, BSA + DPQ and BSA + 2-APB) were incubated the chemicals and then the CHPx stimulants in the control and four were performed in the cells. Scale bar is 20 μm. (**B**) Summarizes the mean fluorescence intensities of Fluo-3 under indicated conditions, from three to four independent experiments, with each experiment examining 10–15 each for each condition. ^a^p < 0.05 indicate significant difference from control. ^b^p < 0.05 indicate significant difference from BSA.

ADPR is produced in nucleus of cells by activation of Poly (ADPR) polymerase-1 (PARP-1) activation^24^. The TRPM2 is stimulated by intracellular ADPR. However, extracellular ADPR cannot pass the cell membrane and the TRPM2 channel is not stimulated by the extracellular ADPR applications^13,22^. DPQ and PJ34 are well-known PARP-1 inhibitors and 2-aminoethoxydiphenyl borate (2-APB) is another non-specific TRPM2 blocker^25,26^. In addition to the ADPR-induced electrophysiology results, we wanted to clarify the role of PARP-1 inhibitors (DPQ and PJ34) and TRPM2 inhibitor (2-APB) on free Ca^2+^ fluorescence intensity in mpkCCD_c14_ cells. Treatment of cells with 2-APB (100 μM) markedly (p < 0.05) suppressed BSA-induced Ca^2+^ fluorescence intensity detected by laser confocal microscope (LSM 800, Zeiss, Ankara, Turkey) analyses (Fig. 5A,B). Treatment with PJ34 (30 μM) or DPQ (10 μM) also markedly (p < 0.05) diminished BSA-induced increase of Ca^2+^ fluorescence intensity (Fig. 5A,B), which is consistent with the involvement of PARP-1 in ADPR-induced TRPM2 activation and nephrotoxicity, as previously described^22,27^.

### Curcumin acted modulator role through inhibition of TRPM2 on albumin-induced apoptosis and mitochondrial oxidative stress in the mpkCCD_c14_ cells

**Figure 6:**
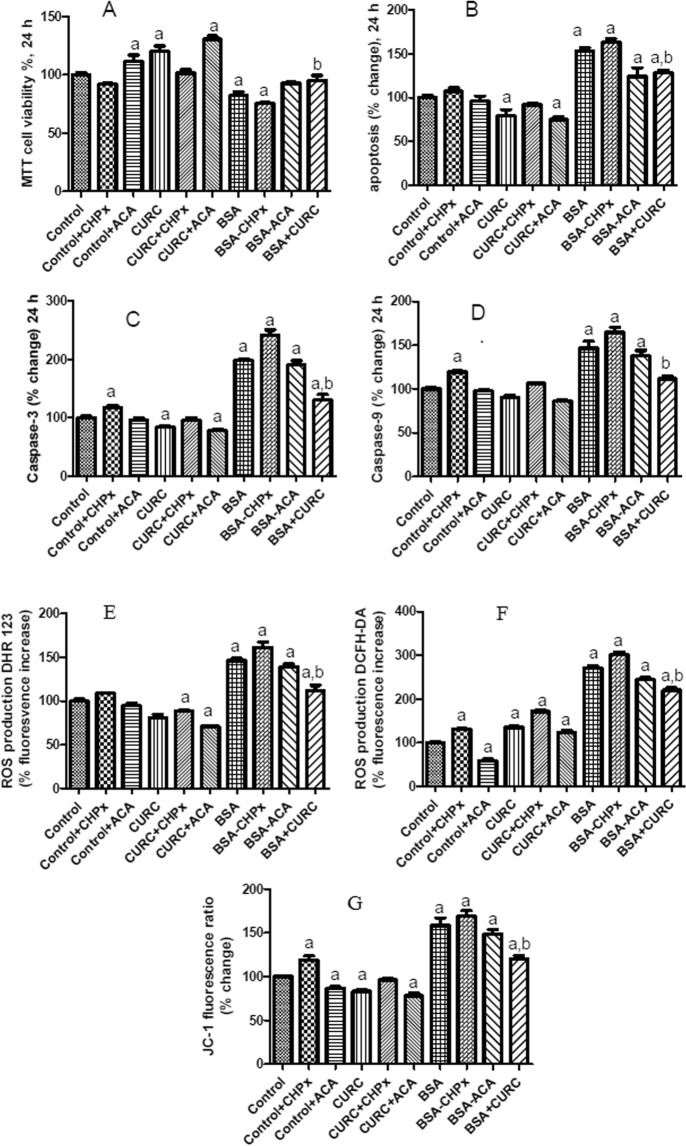
Albumin reduces cell viability and increases apoptosis, mitochondrial membrane depolarization (MMP) and ROS production of mpkCCD_c14_ cells reversed by curcumin (CURC) pretreatment. (**A**–**G**) Cells were treated with albumin (25 mg/ml) and CURC (10 µM), CHPx (1 mM) and ACA (25 µM) or their combinations for 24 h. At this time point (24 h), MTT (**A**) and apoptosis assays were performed. Apoptosis was measured by loss of lipid asymmetry (**B**), caspase-3 (**C**) and caspase-9 activities (**D**) in a microplate reader. In plate reader analyses, two different fluorescent ROS dyes [DHR 123 (**E**) and DCFH-DA (**F**)], and MMP dye JC-1 (**G**) indicated the increase of ROS production and MMP, respectively. The columns represent means with standard deviation (SD) (n = 6 in 2 independent experiments with triplicates). The letters on the columns denote the following: a - significant difference from the control group (p < 0.05). b - significant difference between albumin (BSA) and BSA + CURC treatments (p < 0.05).

Accumulating evidences indicated that the enhance of cytosolic Ca^2+^ concentration stimulates mitochondrial membrane depolarization (MMP) through activation of several cation channels, including TRPM2. In turn, increase of MMP induces activations of two pathways as (1) excessive ROS production and (2) apoptosis through the activation of caspase 9 and 3^28–30^. The modulator role of curcumin on the TRPM2 channel in SH-SY5Y neuroblastoma and hepatocyte cells were recently reported^31,32^. Hence, we searched how BSA influences the cell viability and apoptosis of mpkCCD_c14_ cells in relation to TRPM2 function. At the highest BSA concentration (25 mg/ml) cell viability was significantly decreased (p < 0.05), but curcumin co-treatment diminished the effect of BSA. Both the effects of curcumin and BSA treatments were modulated by the TRPM2 agonist CHPx (1 mM) and the TRPM2 antagonist ACA (25 µM) (Fig. 6A). The roles of curcumin on the induction of apoptosis were assayed by using apoptosis level and caspase (caspase-3 and caspase-9) activity analyses (Fig. 6B–D, respectively). Data analyses of the two complementary assays resulted in two synergic results. High amount of apoptosis, caspase-3 and caspase-9 values were remarked in BSA groups (p < 0.05). Nevertheless, curcumin co-treatment markedly diminished the values to control levels in the cells (p < 0.05). The effects of BSA treatments were also modulated by a TRPM2 agonist, CHPx (1 mM) and a TRPM2 antagonist, ACA (25 µM). The results indicated involvement of BSA-induced TRPM2 activation in the stimulation of the apoptotic pathway.

It is well known that intracellular free Ca^2+^ ion concentration was mainly increased by release of internal Ca^2+^ stores and Ca^2+^ influx from the external side^14^ The intracellular free Ca^2+^ ions are passed into mitochondria^33,34^ and it result in intracellular ROS production and oxidative stress through increase of mitochondrial membrane potential (MMP, Ψ_m_)^35^. The ROS productions (Fig. 6E,F) and MMP levels (Fig. 6G) were markedly (p < 0.05) increased by the BSA incubations (Fig. 6A,B). Again, curcumin co-treatment diminished the effect of albumin through inhibition of TRPM2 activity in the cells (p < 0.05). These results indicated that curcumin diminished albumin-induced mitochondrial oxidative toxicity through decreasing Ca^2+^ influx and TRPM2 channel activity.

### Curcumin reversed the albumin-induced increase of mitochondrial membrane potential and ROS production

In addition to the MMP and ROS microplate reader analyses in the mpkCCD_c14_ cells, we investigated the MMP and ROS image changes in single cells by using the confocal microscopy (LSM 800).

**Figure 7:**
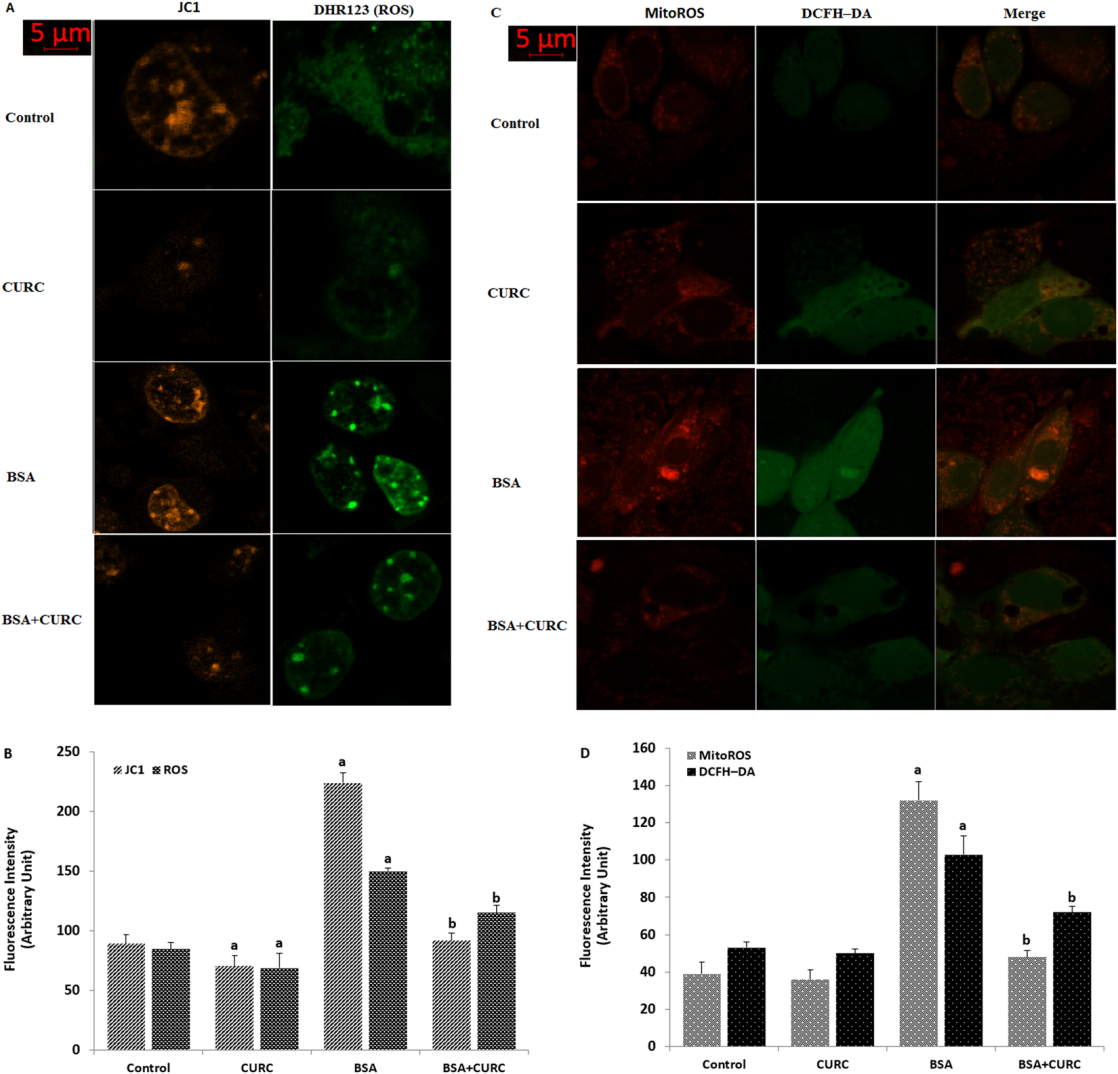
Albumin-induced mitochondrial Ca^2+^ accumulation activates mitochondrial ROS generation in the mpkCCD_c14_ cells. (**A**–**D)**. Cells were treated with albumin (25 mg/ml), CURC (10 µM), CHPx (1 mM) and ACA (25 µM) or their combinations for 24 h. At this time point (24 h), cells were subjected to the JC-1 **(A**,**B)**, DHR 123 (**A**,**B**), MitoTracker Red CM-H2Xros staining (MitoROS) red **(C**,**D)** and DCFH-DA (**C**,**D**) and assays indicating the levels of MMP and ROS production, respectively. Scale bar is 5 μm. (**B**,**D)** are summaries of the mean fluorescence intensity of JC-1, DHR 123, DCFH-DA and MitoROS under indicated conditions from four independent experiments, with each experiment examining 10–15 each for each condition. ^a^p < 0.05 indicate significant difference from control. ^b^p < 0.05 indicate significant difference from BSA.

JC-1 has been used in several cells to analyze MMP in the laser confocal microscope (LSM 800)^36^. Cytosolic ROS imagines were performed by using DHR123 and DCFH-DA fluorescent dyes, although ROS production was imaged by using MitoTracker Red CM-H2Xros staining (MitoROS) in the cells as described in previous studies^25,37^. MMP (Fig. 7A,B), cytosolic (DHR123, Fig. 7A–D) and mitochondrial ROS fluorescence intensities (MitoROS, Fig. 7C,D) were increased in the cells by the BSA treatment. However, curcumin co-treatment attenuated the effect of albumin through inhibition of mitochondrial ROS in the cells. These imaging results further confirmed involvement of curcumin through inhibition of TRPM2 on the albumin-induced mitochondrial activity and oxidative stress in the mpkCCD_c14_ cells.

### Albumin-induced cell death was diminished through inhibition of TRPM2 channel in the kidney cells by curcumin treatment

**Figure 8:**
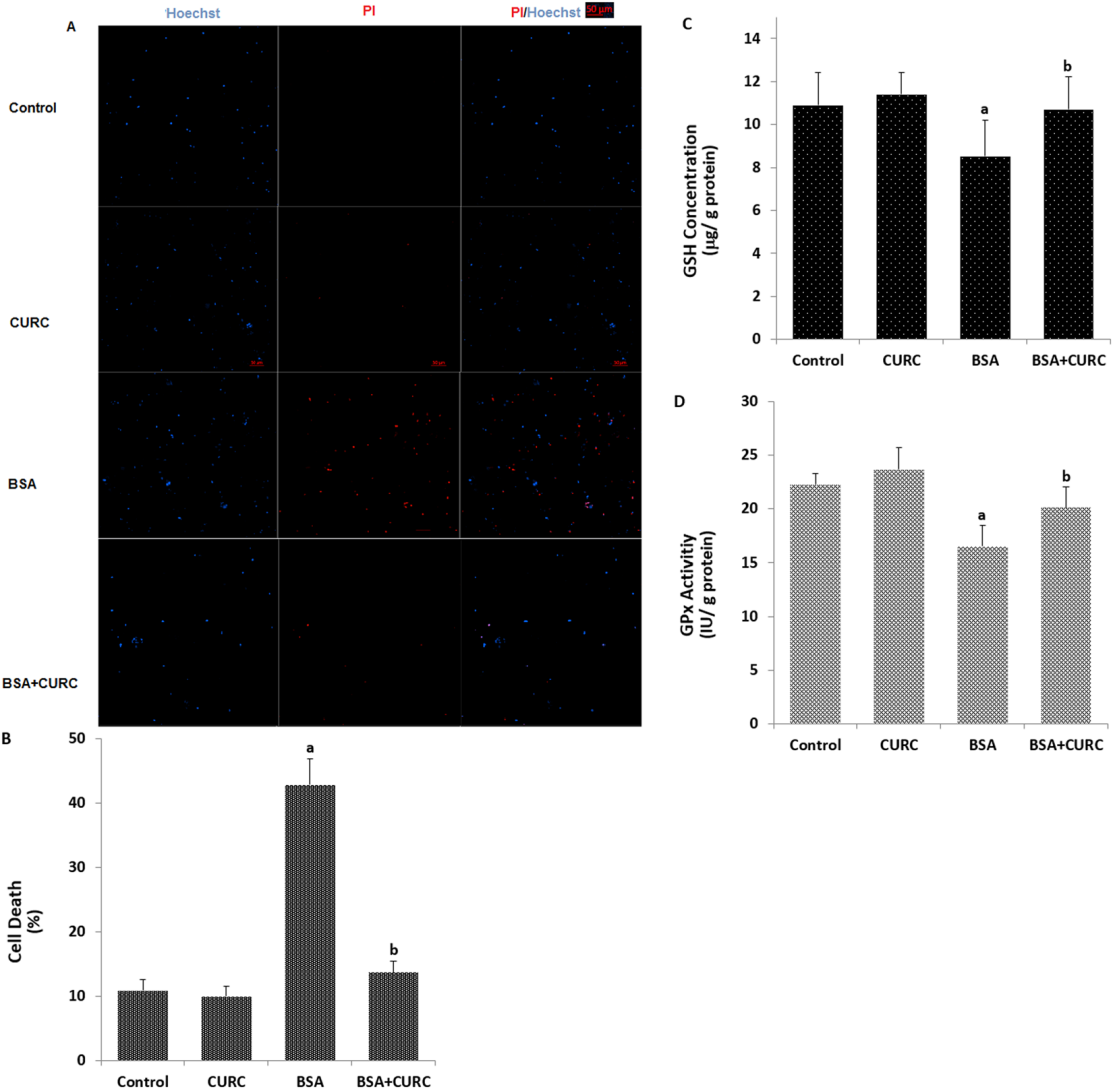
Albumin induces TRPM2 and ROS-dependent kidney (mpkCCD_c14_) cell death through downregulation of reduced glutathione (GSH) and glutathione peroxidase (GPx) activity. (**A**) Representative images show cell death (propidium iodide) and live (Hoechst 33342) staining of mpkCCD_c14_ cells under control conditions or after exposure to albumin (25 mg/ml), curcumin (CURC, 10 µM) and their combinations. Each panel consists of propidium iodide (red) and Hoechst (blue)-stainings showing dead and live cells and merged Hoechst (blue)/PI-stainings showing all and dead cells. Scale bar is 50 μm. (**B**) Summary of the mean percentage of propidium iodide and Hoechst-positive cells under indicated conditions from 6 independent experiments, with each experiment examining 10–15 cells for each condition. The red bars represent the percentage of cell. (**C**,**D**) We also assayed GSH concentration and GPx activity in the four groups by using a spectrophotometer. ^a^p < 0.05 indicate significant difference from control. ^b^p < 0.05 indicate significant difference from BSA.

Oxidative stress dependent activation of TRPM2 increases ROS-induced cell death in different cell types^26–30^. However, involvement of TRPM2 on the albumin (proteinuria)-induced cell death in the mpkCCD_c14_ cells has not been clarified yet. After increasing the apoptosis and ROS levels, we investigated the involvement of TRPM2 channel in the BSA-induced cell death using propidium iodide and Hoechst 33342 fluorescent dyes. We observed that the BSA-induced increase of cell death in the mpkCCD_c14_ cells was completely inhibited by the curcumin treatment (Fig. 8A,B). The results suggest that activation of TRPM2 has a critical role in BSA-induced cell death in kidney cells in the mpkCCD_c14_ cells.

### Curcumin treatment supports glutathione redox system for scavenging albumin-induced excessive ROS production in the kidney cells

ROS are scavenged by antioxidants^24^. Members of thiol redox system such as reduced glutathione (GSH) and glutathione peroxidase (GPx) have a main role in scavenging the ROS in several cells^24^. Curcumin supports the GSH concentration and GPx activity in kidney cells^38,39^. Thiol groups also have a main role in the activation of TRPM2 channels. GSH depletion in neurons induced excessive activation of TRPM2 channels through increased oxidative stress^40–42^. After observing increased levels of ROS, we assume that decreased GSH concentration and GPx activity may induce the activation of the TRPM2 channels in kidney cells. The GSH concentration (Fig. 8C) and GPx (Fig. 8D) activity in the kidney cells were decreased by BSA treatment and levels were increased in the cells treated with curcumin.

## Discussion

Calcium ions (Ca^2+^), acting as signaling molecules in cytosol, ER, and mitochondria, play a fundamental role in the regulation of several biological processes, e.g. metabolism, proliferation, secretion, intercellular communication, and fertilization^14^. Therefore, each cell possesses mechanisms for the precise regulation of Ca^2+^ concentrations in cytoplasm ([Ca^2+^]_i_), ER and mitochondrial matrix^12^. The increased activation of cell membrane Ca^2+^ channels including TRPM2 and intracellular Ca^2+^ channels results in an elevated [Ca^2+^]_i_ concentration^24^. This leads to a mitochondrial Ca^2+^ overload, depolarization of mitochondrial membrane, ROS accumulation, and ATP depletion and, thus, activates the mitochondria-dependent apoptosis^14^. The role of oxidative stress in mitochondrial dysfunction and apoptosis in neurons has been reported in recent studies^28,30^. Apoptosis, caspase 3, caspase 9, PARP-1, MMP and intracellular ROS were decreased after treatment with the TRPM2 blockers (ACA and 2-APB) and PARP-1 (PJ34 and DPQ) inhibitors, while cell viability and antioxidant levels were increased by the treatments^25,26,40–42^. So far, there was no report on the albumin-induced apoptosis, inflammation and TRPM2 channel activation in kidney (mpkCCD_c14_) cells. Here we showed for the first time that albumin-induced increase of apoptosis, caspase 3, caspase 9, MMP, and intracellular ROS in collecting duct cells was attenuated by the treatment with curcumin.

Chronic kidney disease is a public health problem that affects approximately 8–13% of population, independent on sex and age^43^. Oxidative stress and altered Ca^2+^ homeostasis have been implicated in the pathogenesis and progression of CKD^44^. Loss of energy leads to the disruption of Ca^2+^ signaling in ER and mitochondria^19,33,34^. Regarding apoptotic processes, a depletion of Ca^2+^ from ER stores is concomitant with a mitochondrial Ca^2+^ overload^14,19^. Albumin overload has been found to induce endoplasmic reticulum stress and apoptosis in renal proximal tubular cells^45–47^. Excess albumin evoked unfolded protein response and ER stress via elevation of [Ca^2+^]_i_, which led to tubular apoptosis by ATF4-dependent lipocalin 2 modulation^48^. Albumin overload also triggered a stress activated protein kinase, MAPK activation and upregulated MKP-1, an enzyme involved in rapid MAPK dephosphorylation, which might modulate ER stress in renal cells^49^. Moreover, albuminuria induced proinflammatory and profibrotic responses in cortical collecting duct cells. The involvement of the lipocalin 2/NGAL/24p3 receptor (NGAL-R/24p3-R) has been proven^4^. This receptor is expressed in rodent distal nephron, where it mediates protein endocytosis^50^.

In the current study, we showed that TRPM2 activation and mitochondrial ROS production are also involved in the detrimental effect of albuminuria in mpkCCD_c14_ cells. We also demonstrated that curcumin integrating in the cellular membranes effectively reduced the albumin-evoked cytotoxic effects such as intracellular Ca^2+^ overload, NF-kB, cytokine (TNF-α, IL-1β and IL-6) and caspase activations and ROS production. To our knowledge, there is no report of curcumin effects on cortical collecting duct cell function. However, the biological effects of curcumin have already been investigated in several cell lines. For instance, inhibition of amyloid beta-induced cell death and prevention of intracellular Ca^2+^ elevation through inhibition of the NMDA receptors in human neuroblastoma SH-SY5Y cells were reported by curcumin incubation^51^. In this cell line curcumin treatment also reduced oxidative stress evoked by hydrogen peroxide^52^. In addition, apoptosis level and caspase activity of rat renal tubules were inhibited by curcumin treatment through inhibition of nitric oxide synthase^53^. Inhibition of Ca^2+^ mobilization in Jurkat T leukemia cells by curcumin treatment was also reported^54^. In contrary, increases of intracellular ROS production levels, and caspase 3 activation in acute myeloid leukemia^55^ and colorectal cancer cell lines^56^ were reported after treatment with curcumin. According to the above-mentioned reports, effects of curcumin on apoptosis, calcium signaling, cytokine production and oxidative stress are cell specific. This difference in response can be due to the altered Ca^2+^ signaling in tumor cells compared to normal cells^57^.

It is well-documented that PARP-1 has a main role in ROS-mediated TRPM2 gate and cell death in several cell lines^24,25^. As recently reported, ROS stimulates increased TRPM2 channel expression through PARP-1 resulting in cell death induced by ROS^25^. However, curcumin protects against cell death through inhibition of TRPM2 channels in several cell lines^31,32^. It was reported that oxidative stress-induced decrease of GSH concentration and GPx activity, and increase of Ca^2+^ influx in the SH-SY5Y neuroblastoma cells were attenuated by curcumin treatment^52^. Synergic decrease of GSH concentration, GPx activity and PARP-1 expression levels in neurons were induced by oxidative stress^28,30^. In the present study, we observed that BSA-induced cell death (Fig. 8A,B) in mpkCCD_c14_ cells was completely prevented by up-regulation of GSH and GPx activity (Fig. 8C,D) and downregulation of TRPM2 activity by curcumin treatment, indicating that TRPM2 is critical in ROS-induced PARP-1 activation in kidney cells (Supplementary Fig. 3).

The present study provides genetic, physiological and pharmacological evidence to demonstrate the critical role of TRPM2 channel in CKD induced by proteinuria (BSA) at biologically relevant concentrations, in agreement with a recent study showing the role of TRPM2 in the generation of excessive ROS in neuronal cell lines. The results suggest that treatment with curcumin reduces albumin-induced oxidative stress, cell death, and intracellular Ca^2+^ signaling in collecting duct cells (CCD). These findings hold importance and may explain the albumin-induced oxidative injuries in CCD cells, and the renal protective role of curcumin treatment against apoptotic cell death, excessive oxidative stress production, and Ca^2+^ overload. Moreover, TRPM2 channels can be a promising target in the future therapies for CKD.

## Material and Methods

### Reagents and cell lines

Curcumin (CURC), albumin (BSA), dimethyl sulfoxide (DMSO), L-glutamine, Trypsin-EDTA, 3- (4,5-dimethylthiazol-2-yl)-2,5-diphenyltetrazolium bromide (MTT) dye, cumene hydroperoxide (CHPx), ADP-ribose (ADPR), Dihydro-rhodamine 123 (DHR 123) and 2′,7′-dichlorodihydrofluorescein diacetate (DCFH–DA) were purchased from Sigma Aldrich (St. Louis, MO, USA). ACA and 2-APB as two TRPM2 blockers were purchased from Santa Cruz (Istanbul, Turkey). Hoechst 33342, propidium iodide and MitoTracker Red CM-H2Xros (MitoROS) were purchased from Cell Signaling (Istanbul, Turkey).

The murine cortical collecting duct mpkCCD_c14_ cells (obtained from Anatomy, Department of Medicine, Fribourg University, Basel, Switzerland) were cultured in equal volumes of the media Dulbecco’s modified Eagle’s (DMEM) and Ham’s F12 mixed with 60 nmol/l Na^+^ selenate, 5 μg/ml transferrin, 50 nmol/l dexamethasone, 1 nmol/l triiodothyronine, 10 ng/ml epidermal growth factor, 5 μg/ml insulin, 2% fetal bovine serum, and 100 μg/ml penicillin/streptomycin. Cells were grown in a 5% CO_2_/95% air atmosphere at 37 °C and were treated with curcumin (CURC), bovine serum albumin (endotoxine free), TNF, ATP or the specific TRPM2 blocker ACA (25 μM) and agonist CHPx (1 mM) as indicated.

Human embryonic kidney 293 cells (HEK293, purchased from Şap Enstitüsü Ankara, Turkey) were cultured in DMEM as described in a previous study^58^. The cells were analyzed within 24 hours after plating onto the coverslips^13^. The cells were counted by using an automatic cell counter (Casy Modell TT, Roche, Germany).

### Plasmids

To generate cell lines stably expressing the Ca^2+^ indicator proteins, mpkCCD_c14_ cells were infected with lentivirus encoding the gene for CAR-GECO1. The backbone of the lentivirus was produced as described earlier^1,59^. To monitor the NF*-*κB signal transduction pathway *in vitro*, the NF-κB reporter construct from pNF-κB-Luc plasmid (Cat# 219077, Stratagene Inc., La Jolla, CA) was cloned into the lentiviral backbone pLVTHM (purchased from Addgene)^60^. The DNA fragment coding for NF-κB-Luc construct was synthesized by PCR using the following primers: 5′-GAG AGT CGA CCC AAG CTA GGG GAC TTT C-3′ and 5′-GAG AAC TAG TTT TAC AAT TTG GAC TTT CCG C-3′. The amplicon was digested with *SalI* and *SpeI* and inserted into the appropriate sites of pLVTHM to produce the final pLV-NF-κB-Luc plasmid.

To stain specific cell organelles in the immunofluorescence, the following plasmids were used: m-Cherry-ER-3 (endoplasmic reticulum, a gift from Michael Davidson, Addgene plasmid #55041), mito-BFP (mitochondria, a gift from Gia Voeltz^61^, Addgene plasmid #49151), Lamp1-RFP (lysosomes, Addgene plasmid #1817), and mRFP-Clc (clathrin vesicles, Addgene plasmid #14435).

To produce lentivirus, HEK 293 cells were transfected with the expression plasmids pLV-NF-κB-Luc or pLV-CAR-GECO1, the envelope plasmid pMD2G-VSVG (Addgene plasmid #12259) and the packaging plasmid psPAX2 (Addgene plasmid #12260) using calcium phosphate dependent transfection. Supernatants containing the lentivirus were collected after 48 hours and 72 hours, filtered, aliquoted and frozen at −80 °C^62^.

### Measurement of NF-κB and cytokine activities

mpkCCD_c14_ cells were transfected with the NF-κB reporter construct to obtain the NF-κB-luc-expressing mpkCCD_c14_ cells (mpkCCD_c14_^NF-κB-luc^). mpkCCD_c14_^NF-κB-luc^ were seeded in 24-well plates (100,000 cells/well in 500 µl complete medium). On the next day, cells were treated with different concentration of albumin, CURC, ATP and mouse TNF-α solved in 500 µl serum-free mpkCCD_c14_ medium for 6 h. Then the medium was removed. The cells were washed with PBS and lysed for 10 min at room temperature in 100 μl Passive Lysis Buffer (Promega Corp., Madison, WI) per well. The cells were scraped off the wells and lysis was enhanced by several rounds of pipetting up and down. All these steps were performed on ice. The luciferase activity was assessed using 20 μl of the cell lysates and 100 μl of Beetle-Juice from the complete kit (PJK, Kleinblittersdorf, Germany) containing Beetle-Juice buffer, D-luciferin as a substrate and ATP. The enzymatic conversion of luciferin to oxyluciferin through luciferase requires ATP and is associated with the emission of greenish-yellow light between 550–570 nm, which was measured by the TD-20/20 Single-Tube luminometer (Turner BioSystems Inc., Sunnyvale, CA). The measured values were normalized in each experiment to the averaged control value (+500 µl serum-free mpkCCD_c14_ medium). Experiments were repeated three times in triplicates with similar results. Values from one experiment were averaged and statistically evaluated.

To measure IL-1β, IL-6 and TNF-α mpkCCD_c14_ cells were measured according to the protocol provided with the ELISA kit (R&D Systems, Istanbul, Turkey)^20^. Absorbance was detected at 450 nm by the ELISA microplate reader Infinite Pro200. The data were presented as ng/mg protein.

### Curcumin staining

mpkCCD_c14_ cells grown on collagen-coated glass bottom 35 mm dishes (MatTek Corp., Ashland, MA) were transiently transfected using the TransIT*-*2020 transfection reagent according to manufacturer’s instructions (Myrus, Madison, WI) or loaded with Hoechst 33342 dye 10 mg/mL for 20 min at room temperature. Cells were treated with 100 µM curcumin for 5 min in complete medium and washed with buffer solution (DPBS, pH 7.4) that contained 138 mM NaCl, 8 mM Na_2_PO_4_, 2 mM CaCl_2_, 0.5 mM MgCl_2_, 2.7 mM KCl, and 1.6 mM KH_2_PO_4_. Inverted confocal microscope DMI6000 integrated to a Leica TCS-SP5 workstation was used for image acquisition with the following excitation wavelengths and emission bandwidth: 476 nm, 487 nm - 556 nm for CURC; 405 nm, 419–474 nm for mCherry-ER-3, Lamp1-RFP and mRFP-Clc; and 405 nm, 419–474 nm for mito-BFP and Hoechst 33342.

### Ca^2+^ imaging

mpkCCD_c14_ cells expressing CAR-GECO1 grown on collagen-coated glass bottom 35 mm dishes (MatTek Corp., Ashland, MA) were pre-treated with 10 µM CURC in complete medium for 5 min as indicated. After loading, cells were washed with buffer solution (DPBS) used for Ca^2+^-imaging experiments. In the low Ca^2+^ solution, CaCl_2_ was replaced with an equimolar concentration of NaCl. The drugs (THAPS, BSA, ATP, H_2_O_2_) were added to the solutions and remained in the solution until the end of the experiments. We used an inverted confocal microscope DMI6000 integrated to a Leica TCS-SP5 workstation to examine changes in [Ca^2+^]_i_ concentration. To illuminate the Ca^2+^ indicators, we used 476 nm for curcumin and 561 nm for CAR-GECO1. At the confocal microscope, fluorescence emission was recorded at 487–556 nm (CURC) and 584 to 683 nm (CAR-GECO1). Recordings were performed at 37 °C using Tempcontrol 37-*2* digital and a Heating Stage (PeCon GmbH, Erbach, Germany). Fluorescence images for [Ca^2+^]_i_ measurements were collected every 3 s. Circular-shaped regions of interest (ROI) were placed inside the cytoplasmic area of cells. Bleaching correction was carried out, when the baseline was not stable. The relative fluorescent unit (rfu) values were calculated for each cell after background subtraction (fluorescence intensity of regions without cells); fluorescence intensities at each time point (F(t)) were divided by the averaged baseline fluorescence value measured during the non-treatment period (F(0)):

$$rfu(t)=\frac{F(t)}{F(0)}$$

In order to gain insight into evoked Ca^2+^ responses of the entire cell population observed under the microscope, the traces of more than 20 randomly selected cells were averaged:

$$A(t)=\frac{1}{n}\mathop{\sum }\limits_{i=1}^{n}rfu\,(t),$$

where *n* is the number of the selected cells. The integral of the Ca^2+^ signal was calculated as

$${\int }_{t1}^{t0}(A(t)-1)\ast dt,$$

where t0 is the time of the onset of [Ca^2+^]_i_ increment and t1 is the endpoint of the recording period (the time when the signal usually returns to its baseline value). This integral was approximated using the trapezoidal rule. The unit for the Ca^2+^ integrals is rfu*sec. The values of integrals from at least three independent experiments were collected *and were statistically analyzed*.

### Electrophysiology

Whole-cell recordings were performed using an EPC 10 amplifier equipped with a personal computer with Patchmaster software (HEKA, Lamprecht, Germany) at room temperature. The details of standard bath solutions were given in previous studies^28,30^. The holding potential in the mpkCCD_c14_ and HEK293 cells was −60 mV. In the patch-clamp experiment mpkCCD_c14_ and HEK293 cells were perfused with standard bath solution containing ADPR (1 mM in patch pipette) for stimulation or ACA for inhibition (25 μM). The maximal current amplitudes (pA) in a cell were divided by the cell capacitance (pF), a measure of the cell surface. The resulting values represent the current density (pA/pF).

### Cell viability (MTT) assay

To assess albumin’s toxic effects on cell viability, we measured the mitochondrial activity of living mpkCCD_c14_ cells in a microplate reader (Infinite Pro200; Tecan Austria GmbH, Groedig, Austria) at 650 nm by using a 3-(4,5-dimethylthiazol-2-yl)-2,5-diphenyltetrazolium bromide (MTT) quantitative colorimetric assay^52^. The data were presented as percentage relative to the control. The incubation time was 24 hrs. We used concentrations as indicated in Fig. 3D.

### Assay for apoptosis markers

Apoptosis was detected using the APOPercentage Apoptosis Assay (Biocolor, Belfast, Northern Ireland) according to the manufacturer’s instructions. The absorbance of apoptosis dye was measured at 550 nm in a microplate reader (Infinite Pro200). The data were presented as fold increase normalized to control. Activities of caspase-3 and caspase-9 were measured as previously reported with minor modifications^30^. Cleavage of the caspase-3 substrate (AC-DEVD-AMC) and caspase-9 substrate (AC-LEHD-AMC) was measured in a microplate reader (Infinite pro200) with excitation at 360 nm and emission at 460 nm. The data were calculated as fluorescence units/mg protein and presented as fold increase normalized to control.

### Intracellular ROS measurement in the microplate reader

DHR 123 gets fluorescent upon oxidation to yield rhodamine 123 (Rh 123). DCFH-DA yields fluorescent dichlorofluorescein (DCF). Cells were re-suspended in 0.2 ml of extracellular buffer and incubated with DHR 123 (1μl, stock = 20 mM) and 1 μl DCFH-DA (stock = 20 mM) dye solutions for 15 min at room temperature in dark. ROS causes a proportional increase of fluorescence measured in a microplate reader (Infinite Pro200). Excitation was set at 490 nm and emission at 515 nm^28,29^. Data were presented as percental increase normalized to control.

### Mitochondrial membrane potential (MPP) determination in the microplate reader in the mpkCCD_c14_ cells

The mpkCCD_c14_ cells were incubated with 5 μl JC-1 for 15 min at 37 °C as previously described^29^. The lipophilic, cationic dye JC-1 selectively enters into mitochondria and reversibly changes color from red to green, when the mitochondrial potential decreases. The green and red signals were measured by microplate reader Infinite Pro200 at an excitation wavelength of 485 nm and an emission wavelength of 535 nm or at an excitation wavelength of 540 nm and an emission wavelength of 590 nm, respectively. The ratio of green/red fluorescence intensity was calculated and presented as fold increase normalized to control.

### Imaging ROS generation and MMP (JC-1) in the mpkCCD_c14_ cells by laser confocal microscope analyses

Mitochondrial ROS generation in the laser confocal microspore analyses (LSM 800, Zeiss, Ankara, Turkey) was assayed by using MitoTracker Red CM-H2Xros (Life Technologies) fluorescent dye according to manufacturer’s instructions. Intracellular ROS production was monitored by the fluorescent dyes DHR123 and DCFH-DA at 37 °C in dark.

Cells were treated as indicated and then incubated in culture medium containing 100 nM MitoTracker Red CM-H2Xros for 30 min and 1 μM DCFH-DA and DHR123 for 20 min^20,25,26^. Before imaging cells were washed with and maintained in 1xPBS. DHR123 and DCFH-DA were excited with a diode laser at 488 nm. ZEN program was used for analyzing the fluorescence intensity results of each cell. The results were analyzed by using Image J/Imaris software and the mean values were expressed as arbitrary unit.

To image JC-1 in mpkCCD_c14_ cells, cells were incubated with JC-1 (5 μl) fluorescent dye for 15 min at 37 °C in the dark. The samples were then analyzed by the laser confocal microscopy (LSM 800). JC-1 dyes in the cells were excited with a diode laser at 488 nm and an Argon laser at 488 nm^20^. Fluorescence intensity (arbitrary unit) of each cell was recorded by using ZEN program and analyzed by using Image J/Imaris software. Results of staining with JC-1 were expressed as the mean fluorescence intensity in arbitrary units/cell.

### Quantitative PCR using TaqMan probes

RNA was isolated from kidneys and cells according to manufacturer´s instruction (NucleoSpin RNA/protein, Machery & Nagel). 2 ng RNA were reverse transcribed into cDNA using PrimeScript RT Reagent Kit (Takara, RR037A). cDNA was added to Taqman Fast Advanced master mix (Applied Biosystem, 4444556), Euk 18SrRNA (20×) (Applied Biosystem 4319413E) and TRPM2-FAM (mouse: Mm00663098 _m1, human: Hs01066086_m1), and subjected to qPCR.

### Assay of glutathione peroxidase (GSH-Px) activity and reduced glutathione (GSH) level in the mpkCCD_c14_ cells

In the mpkCCD_c14_ cells (10^6^ cells/ml) the level of GSH and the activity of GPx were spectrophotometrically (UV-1800, Shimadzu, Kyoto, Japan) measured at 412 nm by using the methods of Sedlak and Lindsay^62^ and Lawrence and Burk^63^ as described in previous studies^20,30^. The GSH level was expressed as μg/g protein, whereas GPx activity was expressed as IU/g protein. Bradford reagent was used for the total protein assay in the cells at 595 nm by using a spectrophotometer (Shimadzu UV-1800).

### Statistical analysis

Two sample groups were analyzed with Student unpaired t-test. Three and more sample groups were analyzed by one-way ANOVA. If the ANOVA test indicated a statistically significant difference between the groups (*p < 0.05), the data were further analyzed by post hoc tests.

### Compliance with ethical standards

This article does not contain any studies with human participants and experimental animals performed by any of the authors. Cortical collecting duct mpkCCDcl4 cell lines were used in the current study. The procedures in the isolation of the cells were undertaken by the Anatomy Department at the University of Fribourg.

## Supplementary information

Supplementary Figure 1,2,3

Supplementary Dataset 1,2

## Data Availability

All methods in the manuscript were performed in accordance with the relevant guidelines and regulations of Suleyman Demirel University (Isparta, Turkey) and Fribourg University, Fribourg, Switzerland by including a statement in the methods section to this effect. The dataset and analyses were generated in BSN Health, Analyses, Innovation, Consultancy, Organization, Agriculture, Industry and Trade Limited Company, Göller Bölgesi Teknokenti, Isparta, Turkey and are available from the corresponding authors on reasonable request. Graphics in the manuscript were prepared by the corresponding author (Mustafa Nazıroğlu). The authors declare that the main data supporting the findings of this study are available within the article and its Supplementary Information. Extra data that support the findings of this study are available from the corresponding authors upon reasonable request. A reporting summary for this Article is available in the Fig. 9.
