# Supplementary material for: Albumin evokes Ca2+-induced cell oxidative stress and apoptosis through TRPM2 channel in renal collecting duct cells reduced by curcumin
Source: Sci Rep. 2019 Aug 27;9:12403. doi: 10.1038/s41598-019-48716-x (PMC6711968; doi:10.1038/s41598-019-48716-x)
Supplement: Supplementary file 1 — Supplementary Figure 1,2,3 [file 41598_2019_48716_MOESM1_ESM.docx]

**Albumin evokes Ca^2+^-induced cell oxidative stress and apoptosis through TRPM2 channel in renal collecting duct cells reduced by curcumin**

**Mustafa Nazıroğlu^1,2,3^, Bilal Çiğ^2,3^, Yener Yazğan^2,3^, Gerburg K. Schwaerzer^4^, Franziska Theilig^4,5^, László Pecze^5,6^**

^1^Neuroscience Research Center, Suleyman Demirel University, Isparta, Turkey

^2^Department of Biophysics, Faculty of Medicine, Suleyman Demirel University, Isparta, Turkey

^3^Department of Neuroscience, Health Science Institute, Suleyman Demirel University, Isparta, Turkey

^4^Institute of Anatomy, Christian-Albrechts-University of Kiel, Kiel, Germany.

^5^Anatomy, Department of Medicine, University of Fribourg, Fribourg, Switzerland

^6^Independent Scientist, Neuchhatel, Switzerland

ABC

DEF

**Supplementary Fig.1 Amplitudes and frequencies (A, B, C)**. Statistical comparison of the amplitudes of the evoked Ca^2+^ responses with/without curcumin pretreatment **(D, E, F**). Statistical comparison of the amplitudes of the evoked Ca^2+^ responses with/without curcumin pretreatment. Compounds were administered in the following concentrations: BSA 25 ng/ml , ATP 5µM, H_2_O_2_ 0.15% CURC pre-treatment 10 µM, 5 min.


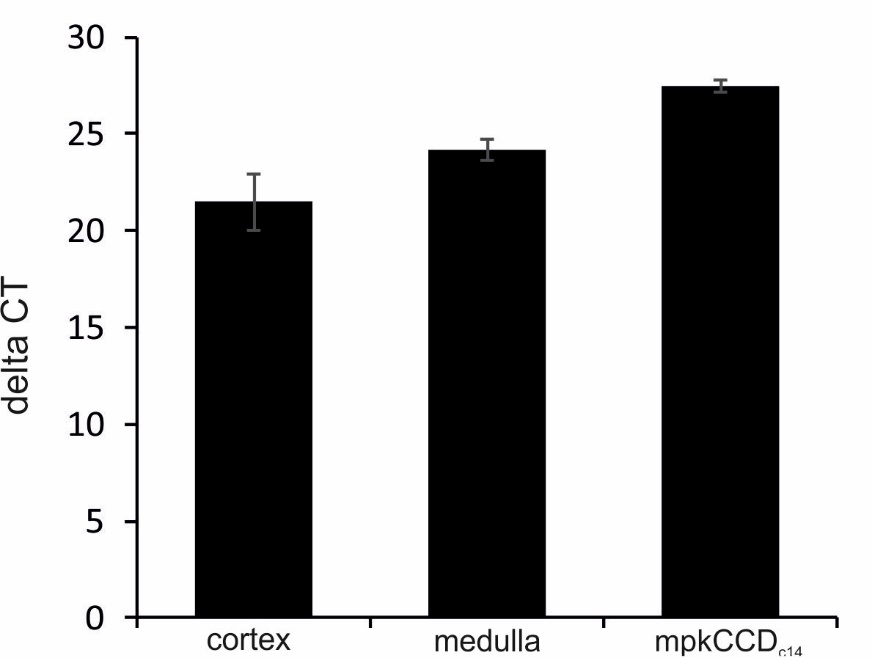


**Supplementary Fig.2**. TRPM2 mRNA expression in renal cortex, medulla and in mpkCCD_c14_ cells. Delta CT values are shown and were calculated from CT vlues of TRPM2 – CT values of 18SrRNA, n = 3 . TRPM2 mRNA is found in the kidney cortex, medulla and mpkCCD_c14_ cells.


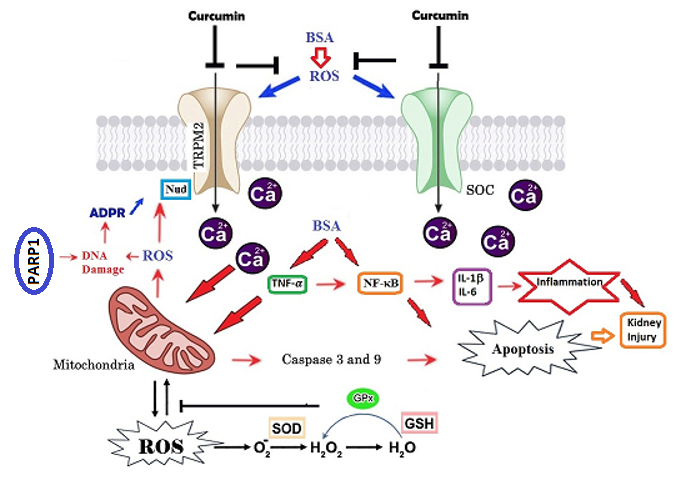


**Supplementary Fig. 3. Possible protective action of curcumin on proteinuria (albumin)-induced apoptosis and inflammation through modulation of TRPM2 signaling in the renal collecting duct cells**. ROS are produced in physiological and pathophysiological functions of mitochondria. Oxidative stress induces ADP-ribose (ADPR) production and DNA damage through activation of PARP1enyzme. The ADPR and ROS contact with the Nudix (Nud) domain of TRPM2 leading to huge increases in intracellular Ca^2+^ concentration, BSA increases the production of ROS, promotes the Ca^2+^ influx via TRPM2, and increases the mitochondrial membrane potential, which further increases the ROS production. In addition, albumin induces the activation of NF-κB and the concentration of IL-1β and IL-6, which results in inflammation. However, it induces decrease of reduced glutathione (GSH) and glutathione peroxidase (GPx). Treatment with curcumin reduces the production of ROS, the TRPM2-dependent Ca^2+^ influx and the activation of NF-κB signaling. Therefore, collecting duct cells treated with curcumin showed a higher viability and reduced apoptosis.
