# Supplementary material for: Albumin evokes Ca2+-induced cell oxidative stress and apoptosis through TRPM2 channel in renal collecting duct cells reduced by curcumin
Source: Sci Rep. 2019 Aug 27;9:12403. doi: 10.1038/s41598-019-48716-x (PMC6711968; doi:10.1038/s41598-019-48716-x)
Supplement: Supplementary file 2 — Supplementary Dataset 1,2 [file 41598_2019_48716_MOESM2_ESM.docx]

**Albumin evokes Ca^2+^-induced cell oxidative stress and apoptosis through TRPM2 channel in renal collecting duct cells reduced by curcumin**

**Mustafa Nazıroğlu^1,2,3^, Bilal Çiğ^2,3^, Yener Yazğan^2,3^, Gerburg K. Schwaerzer^4^, Franziska Theilig^4,5^, László Pecze^5,6^**

^1^Neuroscience Research Center, Suleyman Demirel University, Isparta, Turkey

^2^Department of Biophysics, Faculty of Medicine, Suleyman Demirel University, Isparta, Turkey

^3^Department of Neuroscience, Health Science Institute, Suleyman Demirel University, Isparta, Turkey

^4^Institute of Anatomy, Christian-Albrechts-University of Kiel, Kiel, Germany.

^5^Anatomy, Department of Medicine, University of Fribourg, Fribourg, Switzerland

^6^Independent Scientist, Neuchhatel, Switzerland

**Supplementary Material**

1. **Frequency and ampliitude analys of the evoked Ca^2+^ oscillations**

Time-lapse recordings from individual cells showing Ca^2+^ oscillations have been collected and the data was analysed by the following MATLAB program :

clear all

close all

% read the csv file

numA = csvread('C:\Users\Pecze Laszlo\Documents\Everything\Laci\ertekeles48\CCDH2O2CURCexp5.csv', 3,0)

mes_last= size(numA, 2)

background=15 %min(numA(:, mes_last))

%background substraction

numB=numA-background

%normalisation

num=numB./numB(1,:)

ttime= numA(:, 1)' %time vector (' = transpose)

mes_length= size(num, 2)-1

for i=2:mes_length

mes=num(:,i)' % one measurement

figure (1)

plot (ttime, mes, '-o')

hold on

%BLEACHING CORRECTION

opol = 6; %order of polinomial

[p,s,mu] = polyfit(ttime (mes<1.1),mes(mes<1.1),opol);

ydev = polyval(p,ttime,[],mu); % polinomvalues

corrmes = mes - ydev +1; % correction

plot (ttime, corrmes,'-ored')

hold off

legend('orginal','corrected')

xlabel('time (s)')

ylabel('rfu')

%PEAK ANALYSIS

figure (2)

findpeaks(corrmes,ttime, 'MinPeakProminence',0.25)

[pks, locs]=findpeaks(corrmes,ttime, 'MinPeakProminence',0.25)

peakInterval = diff(locs);

freq=1/(mean(peakInterval)) %average frequency

amplitude=mean (pks)-1 %average amplitude

title(['freqency=' num2str(freq) ' Hz'])

xlabel('time (s)')

ylabel('rfu')

AMP_LIST(i-1)=amplitude

FREQ_LIST(i-1)=freq

end

figure(3)

hist(AMP_LIST)

title('Amplitude')

figure(4)

hist(FREQ_LIST)

title('Frequency')

filename = 'C:\Users\Pecze Laszlo\Documents\Everything\Laci\ertekeles48\evaluation2.xlsx';

A = [AMP_LIST', FREQ_LIST'];

sheet = 6

xlRange = 'E2';

xlswrite(filename,A,sheet,xlRange)

Frequenc and Amplitude measurements from different experiments were pooled and descriptive statistics was performed See Supplementary Table 1 and Table.2. Difference in frequencies and amplitudes were.analysed by nonparametric Mann–Whitney *U* test. A significance level less than 0.05 has been considred as significant.

| **AMPLITUDE** | **BSA** | **BSA+CURC** | **ATP** | **ATP+CURC** | **H_2_O_2_** | **H_2_O_2_+CURC** |
| --- | --- | --- | --- | --- | --- | --- |
| Number of cellhs | 140 | 77 | 201 | 203 | 111 | 116 |
| Minimum | 0.1292 | 0.1307 | 0.1327 | 0.4945 | 0.2402 | -0.02465 |
| 25% Percentile | 1.159 | 0.7712 | 1.077 | 0.9199 | 2.184 | 0.5534 |
| Median | 1.797 | 1.658 | 2.154 | 1.367 | 2.77 | 1.391 |
| 75% Percentile | 2.292 | 2.283 | 3.64 | 2.364 | 3.734 | 2.4 |
| Maximum | 4.855 | 3.687 | 21.27 | 6.558 | 8.145 | 7.034 |
| Mean | 1.795 | 1.556 | 3.311 | 1.784 | 2.973 | 1.658 |
| Std. Deviation | 0.924 | 0.9619 | 3.729 | 1.188 | 1.569 | 1.333 |
| Std. Error | 0.07809 | 0.1096 | 0.263 | 0.08337 | 0.1489 | 0.1238 |
| Lower 95% CI | 1.641 | 1.337 | 2.793 | 1.619 | 2.678 | 1.413 |
| Upper 95% CI | 1.95 | 1.774 | 3.83 | 1.948 | 3.268 | 1.903 |

**Table 1.** Descriptive statistics of the amplitudes of the evoked Ca^2+^ responses with/without curcumin pretreatment. Compounds were administered in the following concentrations: BSA 25 ng/ml , ATP 5µM, H_2_O_2_ 0.15% CURC pre-treatment 10 µM, 5 min.

| **FREQUENCY** | **BSA** | **BSA+CURC** | **ATP** | **ATP+CURC** | **H**_2_O_2_ | **H**_2_O_2_+CURC |
| --- | --- | --- | --- | --- | --- | --- |
| Number of values | 135 | 63 | 176 | 137 | 74 | 105 |
| Minimum | 0.003401 | 0.002469 | 0.002451 | 0.002237 | 0.002252 | 0.003584 |
| 25% Percentile | 0.01274 | 0.008333 | 0.02074 | 0.008063 | 0.01797 | 0.01771 |
| Median | 0.01987 | 0.01274 | 0.03333 | 0.01414 | 0.02761 | 0.02519 |
| 75% Percentile | 0.03263 | 0.01961 | 0.05 | 0.02295 | 0.03302 | 0.03638 |
| Maximum | 0.0625 | 0.08333 | 0.1667 | 0.08333 | 0.05983 | 0.08333 |
| Mean | 0.02283 | 0.01932 | 0.03919 | 0.01686 | 0.02674 | 0.02728 |
| Std. Deviation | 0.01298 | 0.01969 | 0.02644 | 0.01254 | 0.01154 | 0.01421 |
| Std. Error | 0.001117 | 0.002481 | 0.001993 | 0.001071 | 0.001342 | 0.001387 |
| Lower 95% CI | 0.02062 | 0.01436 | 0.03526 | 0.01474 | 0.02407 | 0.02453 |
| Upper 95% CI | 0.02504 | 0.02428 | 0.04312 | 0.01898 | 0.02942 | 0.03003 |

Table 2. Descriptive statistics of the amplitudes of the evoked Ca^2+^ responses with/without curcumin pretreatment. Compounds were administered in the following concentrations: BSA25 ng/ml , ATP 5µM, H_2_O_2_ 0.15% CURC pre-treatment 10 µM, 5 min.

**2. Image overlap analysis**

**A**: Curcumin staining does not co-localize with nuclei: curcumin (green), nucleus (blue).

Image A: Clipboard (green)

Image B: Clipboard (blue)

Pearson's Coefficient:

r=-0.185

Overlap Coefficient:

r=0.309

r^2=k1xk2:

k1=0.18

k2=0.529

Using thresholds (thrA=103 and thrB=98)

Overlap Coefficient:

r=0.958

r^2=k1xk2:

k1=0.995

k2=0.923

Manders' Coefficients (original):

M1=0.233 (fraction of A overlapping B)

M2=1.0 (fraction of B overlapping A)

Manders' Coefficients (using threshold value of 103 for imgA and 98 for imgB):

M1=0.038 (fraction of A overlapping B)

M2=0.103 (fraction of B overlapping A)

Costes' automatic threshold set to 255 for imgA & 255 for imgB

Pearson's Coefficient:

r=0.0 (1.0 below thresholds)

M1=0.0 & M2=0.0

Van Steensel's Cross-correlation Coefficient between Clipboard (green) and Clipboard (blue):

CCF min.: -0.193 (obtained for dx=1) CCF max.: 0.032 (obtained for dx=-20)

Results for fitting CCF on a Gaussian (CCF=a+(b-a)exp(-(xshift-c)^2/(2d^2))):

Formula: y = a + (b-a)*exp(-(x-c)*(x-c)/(2*d*d))

Status: Success

Number of completed minimizations: 2

Number of iterations: 115 (max: 6000)

Time: 3 ms

Sum of residuals squared: 0.12750

Standard deviation: 0.056458

R^2: 0.40226

Parameters:

a = -0.10078

b = 0.029275

c = -17.80740

d = 5.21671

FWHM=12.284 pixels

Cytofluorogram's parameters:

a: -0.279

b: 48.692

Correlation coefficient: -0.185

Li's Intensity correlation coefficient:

ICQ: -0.03436315525995853

Costes' randomization based colocalization:

Parameters: Nb of randomization rounds: 1000, Resolution (bin width): 0.001

r (original)=-0.186

r (randomized)=0.0±0.008 (calculated from the fitted data)

P-value=0.0% (calculated from the fitted data)

Results for fitting the probability density function on a Gaussian (Probability=a+(b-a)exp(-(R-c)^2/(2d^2))):

Formula: y = a + (b-a)*exp(-(x-c)*(x-c)/(2*d*d))

Status: Success

Number of completed minimizations: 2

Number of iterations: 119 (max: 6000)

Time: 5 ms

Sum of residuals squared: 0.00086653

Standard deviation: 0.0044891

R^2: 0.93421

Parameters:

a = 0.00062873

b = 0.049404

c = -4.25431E-5

d = 0.0080023

FWHM=0.018

Colocalization based on distance between centres of mass

Threshold for Image A=103; Image B=98

Particles size between 0 & 55932

Image A: 5 centre(s) colocalizing out of 202

Image B: 5 centre(s) colocalizing out of 87

Colocalization based on centres of mass-particles coincidence

Threshold for Image A=103; Image B=98

Particles size between 0 & 55932

Image A: 13 centre(s) colocalizing out of 202

Image B: 4 centre(s) colocalizing out of 87

**B**: Curcumin staining does not co-localize with mitochondria: curcumin (green), mitochondria (blue).

Image A: Clipboard (green)

Image B: Clipboard (blue)

Pearson's Coefficient:

r=0.27

Overlap Coefficient:

r=0.343

r^2=k1xk2:

k1=0.161

k2=0.731

Using thresholds (thrA=64 and thrB=81)

Overlap Coefficient:

r=0.918

r^2=k1xk2:

k1=1.301

k2=0.648

Manders' Coefficients (original):

M1=0.326 (fraction of A overlapping B)

M2=1.0 (fraction of B overlapping A)

Manders' Coefficients (using threshold value of 64 for imgA and 81 for imgB):

M1=0.096 (fraction of A overlapping B)

M2=0.82 (fraction of B overlapping A)

Costes' automatic threshold set to 47 for imgA & 6 for imgB

Pearson's Coefficient:

r=-0.051 (0.0 below thresholds)

M1=0.731 & M2=0.976

Van Steensel's Cross-correlation Coefficient between Clipboard (green) and Clipboard (blue):

CCF min.: 0.036 (obtained for dx=-20) CCF max.: 0.27 (obtained for dx=0)

Results for fitting CCF on a Gaussian (CCF=a+(b-a)exp(-(xshift-c)^2/(2d^2))):

Formula: y = a + (b-a)*exp(-(x-c)*(x-c)/(2*d*d))

Status: Success

Number of completed minimizations: 2

Number of iterations: 99 (max: 6000)

Time: 2 ms

Sum of residuals squared: 0.012750

Standard deviation: 0.017853

R^2: 0.92071

Parameters:

a = 0.024090

b = 0.23750

c = 2.31389

d = 10.83741

FWHM=25.52 pixels

Cytofluorogram's parameters:

a: 0.176

b: -1.322

Correlation coefficient: 0.27

Li's Intensity correlation coefficient:

ICQ: 0.18198884359579492

Costes' randomization based colocalization:

Parameters: Nb of randomization rounds: 1000, Resolution (bin width): 0.001

r (original)=0.27

r (randomized)=0.0±0.007 (calculated from the fitted data)

P-value=100.0% (calculated from the fitted data)

Results for fitting the probability density function on a Gaussian (Probability=a+(b-a)exp(-(R-c)^2/(2d^2))):

Formula: y = a + (b-a)*exp(-(x-c)*(x-c)/(2*d*d))

Status: Success

Number of completed minimizations: 2

Number of iterations: 117 (max: 6000)

Time: 20 ms

Sum of residuals squared: 0.0013746

Standard deviation: 0.0055269

R^2: 0.90952

Parameters:

a = 0.0015490

b = 0.050870

c = 0.00037016

d = 0.0075508

FWHM=0.017

Colocalization based on distance between centres of mass

Threshold for Image A=64; Image B=81

Particles size between 0 & 55932

Image A: 4 centre(s) colocalizing out of 57

Image B: 4 centre(s) colocalizing out of 17

Colocalization based on centres of mass-particles coincidence

Threshold for Image A=64; Image B=81

Particles size between 0 & 55932

Image A: 5 centre(s) colocalizing out of 57

Image B: 5 centre(s) colocalizing out of 17

**C:** Curcumin is partially present on endoplasmic membranes: Curcumin (green), endoplasmic reticulum (red)

Image A: Clipboard (red)

Image B: Clipboard (green)

Pearson's Coefficient:

r=0.636

Overlap Coefficient:

r=0.793

r^2=k1xk2:

k1=0.998

k2=0.629

Using thresholds (thrA=85 and thrB=82)

Overlap Coefficient:

r=0.953

r^2=k1xk2:

k1=0.941

k2=0.966

Manders' Coefficients (original):

M1=0.999 (fraction of A overlapping B)

M2=0.999 (fraction of B overlapping A)

Manders' Coefficients (using threshold value of 85 for imgA and 82 for imgB):

M1=0.889 (fraction of A overlapping B)

M2=0.495 (fraction of B overlapping A)

Costes' automatic threshold set to 2 for imgA & 42 for imgB

Pearson's Coefficient:

r=0.579 (0.0 below thresholds)

M1=0.999 & M2=0.852

Van Steensel's Cross-correlation Coefficient between Clipboard (red) and Clipboard (green):

CCF min.: 0.334 (obtained for dx=20) CCF max.: 0.636 (obtained for dx=0)

Results for fitting CCF on a Gaussian (CCF=a+(b-a)exp(-(xshift-c)^2/(2d^2))):

Formula: y = a + (b-a)*exp(-(x-c)*(x-c)/(2*d*d))

Status: Success

Number of completed minimizations: 2

Number of iterations: 99 (max: 6000)

Time: 5 ms

Sum of residuals squared: 0.026981

Standard deviation: 0.025972

R^2: 0.87115

Parameters:

a = 0.37707

b = 0.57030

c = -0.60623

d = 4.53246

FWHM=10.673 pixels

Cytofluorogram's parameters:

a: 0.64

b: 41.086

Correlation coefficient: 0.636

Li's Intensity correlation coefficient:

ICQ: 0.2034975581729388

Costes' randomization based co.
